# Locus coeruleus signal intensity and emotion regulation in agitation in Alzheimer’s disease

**DOI:** 10.1093/braincomms/fcae457

**Published:** 2024-12-17

**Authors:** Kathy Y Liu, Matthew J Betts, Dorothea Hämmerer, Emrah Düzel, Mara Mather, Jonathan P Roiser, Anja Schneider, Annika Spottke, Ayda Rostamzadeh, Björn H Schott, Boris-Stephan Rauchmann, Christoph Laske, Daniel Janowitz, Eike J Spruth, Ersin Ersözlü, Falk Lüsebrink, Frank Jessen, Ingo Frommann, Ingo Kilimann, Jens Wiltfang, Johanna Brustkern, Josef Priller, Julian Hellman-Regen, Katharina Buerger, Klaus Fliessbach, Klaus Scheffler, Luca Kleineidam, Melina Stark, Michael Ewers, Michael Wagner, Oliver Peters, Peter Dechent, Robert Perneczky, Sebastian Sodenkamp, Stefan Hetzer, Stefan Teipel, Wenzel Glanz, Robert Howard

**Affiliations:** Division of Psychiatry, University College London, London W1T 7NF, UK; German Center for Neurodegenerative Diseases (DZNE), 39120 Magdeburg, Germany; Institute of Cognitive Neurology and Dementia Research (IKND), Otto-von-Guericke University, 39120 Magdeburg, Germany; CBBS Center for Behavioral Brain Sciences, 39106 Magdeburg, Germany; German Center for Neurodegenerative Diseases (DZNE), 39120 Magdeburg, Germany; Institute of Cognitive Neurology and Dementia Research (IKND), Otto-von-Guericke University, 39120 Magdeburg, Germany; CBBS Center for Behavioral Brain Sciences, 39106 Magdeburg, Germany; Department of Psychology, University of Innsbruck, 6020 Innsbruck, Austria; Institute of Cognitive Neuroscience, University College London, London WC1N 3AZ, UK; German Center for Neurodegenerative Diseases (DZNE), 39120 Magdeburg, Germany; Institute of Cognitive Neurology and Dementia Research (IKND), Otto-von-Guericke University, 39120 Magdeburg, Germany; CBBS Center for Behavioral Brain Sciences, 39106 Magdeburg, Germany; Leonard Davis School of Gerontology, University of Southern California, Los Angeles, CA 90089, USA; Institute of Cognitive Neuroscience, University College London, London WC1N 3AZ, UK; German Center for Neurodegenerative Diseases (DZNE), 53127 Bonn, Germany; Department for Cognitive Disorders and Old Age Psychiatry, University Hospital Bonn, 53127 Bonn, Germany; German Center for Neurodegenerative Diseases (DZNE), 53127 Bonn, Germany; Department of Neurology, University of Bonn, 53127 Bonn, Germany; Department of Psychiatry, University of Cologne, Medical Faculty, 50924 Cologne, Germany; CBBS Center for Behavioral Brain Sciences, 39106 Magdeburg, Germany; German Center for Neurodegenerative Diseases (DZNE), 37075 Goettingen, Germany; Department of Psychiatry and Psychotherapy, University Medical Center Goettingen, University of Goettingen, 37075 Goettingen, Germany; Department of Behavioral Neurology, Leibniz Institute for Neurobiology, 39118 Magdeburg, Germany; Department of Psychiatry and Psychotherapy, University Hospital, 80336 Munich, Germany; Sheffield Institute for Translational Neuroscience (SITraN), University of Sheffield, Sheffield S10 2HQ, UK; Department of Neuroradiology, University Hospital LMU, 81377 Munich, Germany; German Center for Neurodegenerative Diseases (DZNE), 72076 Tübingen, Germany; Section for Dementia Research, Hertie Institute for Clinical Brain Research and Department of Psychiatry and Psychotherapy, University of Tübingen, 72076 Tübingen, Germany; Institute for Stroke and Dementia Research (ISD), University Hospital, 81377 Munich, Germany; German Center for Neurodegenerative Diseases (DZNE), 10117 Berlin, Germany; Department of Psychiatry and Psychotherapy, Charité, 10117 Berlin, Germany; German Center for Neurodegenerative Diseases (DZNE), 10117 Berlin, Germany; Charité—Universitätsmedizin Berlin, corporate member of Freie Universität Berlin and Humboldt-Universität zu Berlin-Institute of Psychiatry and Psychotherapy, 10117 Berlin, Germany; German Center for Neurodegenerative Diseases (DZNE), 39120 Magdeburg, Germany; German Center for Neurodegenerative Diseases (DZNE), 53127 Bonn, Germany; Department of Neurology, University of Bonn, 53127 Bonn, Germany; Excellence Cluster on Cellular Stress Responses in Aging-Associated Diseases (CECAD), University of Cologne, 50931 Köln, Germany; German Center for Neurodegenerative Diseases (DZNE), 53127 Bonn, Germany; Department for Cognitive Disorders and Old Age Psychiatry, University Hospital Bonn, 53127 Bonn, Germany; German Center for Neurodegenerative Diseases (DZNE), 18147 Rostock, Germany; Department of Psychosomatic Medicine, Rostock University Medical Center, 18147 Rostock, Germany; German Center for Neurodegenerative Diseases (DZNE), 37075 Goettingen, Germany; Department of Psychiatry and Psychotherapy, University Medical Center Goettingen, University of Goettingen, 37075 Goettingen, Germany; Neurosciences and Signaling Group, Institute of Biomedicine (iBiMED), Department of Medical Sciences, University of Aveiro, 3810-193 Aveiro, Portugal; German Center for Neurodegenerative Diseases (DZNE), 53127 Bonn, Germany; German Center for Neurodegenerative Diseases (DZNE), 10117 Berlin, Germany; Department of Psychiatry and Psychotherapy, Charité, 10117 Berlin, Germany; Department of Psychiatry and Psychotherapy, School of Medicine, Technical University of Munich, 81675 Munich, Germany; University of Edinburgh and UK DRI, Edinburgh EH16 4SB, UK; German Center for Neurodegenerative Diseases (DZNE), 10117 Berlin, Germany; Department of Psychiatry and Neurosciences, Charité Universitätsmedizin Berlin, 12203 Berlin, Germany; German Center for Mental Health (DZPG), partner site Berlin, 10117 Berlin, Germany; Institute for Stroke and Dementia Research (ISD), University Hospital, 81377 Munich, Germany; German Center for Neurodegenerative Diseases (DZNE, Munich), 81377 Munich, Germany; German Center for Neurodegenerative Diseases (DZNE), 53127 Bonn, Germany; Department for Cognitive Disorders and Old Age Psychiatry, University Hospital Bonn, 53127 Bonn, Germany; Department for Biomedical Magnetic Resonance, University of Tübingen, 72076 Tübingen, Germany; German Center for Neurodegenerative Diseases (DZNE), 53127 Bonn, Germany; Department for Cognitive Disorders and Old Age Psychiatry, University Hospital Bonn, 53127 Bonn, Germany; German Center for Neurodegenerative Diseases (DZNE), 53127 Bonn, Germany; Department for Cognitive Disorders and Old Age Psychiatry, University Hospital Bonn, 53127 Bonn, Germany; German Center for Neurodegenerative Diseases (DZNE, Munich), 81377 Munich, Germany; German Center for Neurodegenerative Diseases (DZNE), 53127 Bonn, Germany; Department for Cognitive Disorders and Old Age Psychiatry, University Hospital Bonn, 53127 Bonn, Germany; German Center for Neurodegenerative Diseases (DZNE), 10117 Berlin, Germany; Charité—Universitätsmedizin Berlin, corporate member of Freie Universität Berlin and Humboldt-Universität zu Berlin-Institute of Psychiatry and Psychotherapy, 10117 Berlin, Germany; MR-Research in Neurosciences, Department of Cognitive Neurology, Georg-August-University Goettingen, 37075 Goettingen, Germany; Department of Psychiatry and Psychotherapy, University Hospital, 80336 Munich, Germany; German Center for Neurodegenerative Diseases (DZNE, Munich), 81377 Munich, Germany; Munich Cluster for Systems Neurology (SyNergy) Munich, 81377 Munich, Germany; Ageing Epidemiology Research Unit (AGE), School of Public Health, Imperial College London, London W6 8RP, UK; German Center for Neurodegenerative Diseases (DZNE), 72076 Tübingen, Germany; Department of Psychiatry and Psychotherapy, University of Tübingen, 72076 Tübingen, Germany; Berlin Center for Advanced Neuroimaging, Charité—Universitätsmedizin Berlin, 10117 Berlin, Germany; German Center for Neurodegenerative Diseases (DZNE), 18147 Rostock, Germany; Department of Psychosomatic Medicine, Rostock University Medical Center, 18147 Rostock, Germany; German Center for Neurodegenerative Diseases (DZNE), 39120 Magdeburg, Germany; Division of Psychiatry, University College London, London W1T 7NF, UK

**Keywords:** locus coeruleus, Alzheimer, autonomic, emotion regulation, agitation

## Abstract

Hyperphosphorylated tau accumulation is seen in the noradrenergic locus coeruleus from the earliest stages of Alzheimer’s disease onwards and has been associated with symptoms of agitation. It is hypothesized that compensatory locus coeruleus-noradrenaline system overactivity and impaired emotion regulation could underlie agitation propensity, but to our knowledge this has not previously been investigated. A better understanding of the neurobiological underpinnings of agitation would help the development of targeted prevention and treatment strategies.

Using a sample of individuals with amnestic mild cognitive impairment and probable mild Alzheimer’s disease dementia from the German Center for Neurodegenerative Diseases (DZNE)-Longitudinal Cognitive Impairment and Dementia (DELCODE) study cohort (*N* = 309, aged 67–96 years, 51% female), we assessed cross-sectional relationships between a latent factor representing the functional integrity of an affect-related executive regulation network and agitation point prevalence and severity scores. In a subsample of individuals with locus coeruleus MRI imaging data (*N* = 37, aged 68–93 years, 49% female), we also investigated preliminary associations between locus coeruleus MRI contrast ratios (a measure of structural integrity, whole or divided into rostral, middle, and caudal thirds) and individual affect-related regulation network factor scores and agitation measures. Regression models controlled for effects of age and clinical disease severity and, for models including resting-state functional MRI connectivity variables, grey matter volume and education years.

Agitation point prevalence showed a positive relationship with a latent factor representing the functional integrity (and a negative relationship with a corresponding structural measure) of the affect-related executive regulation network. Locus coeruleus MRI contrast ratios were positively associated with agitation severity (but only for the rostral third, in *N* = 13) and negatively associated with the functional affect-related executive regulation latent factor scores. Resting-state functional connectivity between a medial prefrontal cortex region and the left amygdala was related to locus coeruleus MRI contrast ratios.

These findings implicate the involvement of locus coeruleus integrity and emotion dysregulation in agitation in Alzheimer’s disease and support the presence of potential compensatory processes. At the neural level, there may be a dissociation between mechanisms underlying agitation risk *per se* and symptom severity. Further studies are needed to replicate and extend these findings, incorporating longitudinal designs, measures of autonomic function and non-linear modelling approaches to explore potential causal and context-dependent relationships across Alzheimer’s disease stages.

## Teaser

Liu *et al*. report agitation in early Alzheimer’s disease was positively related to a functional measure (and negatively related to a structural measure) of affect-related executive regulation, and symptom severity was associated with higher locus coeruleus MRI contrast ratios. Early noradrenergic system-related compensatory processes may underlie agitation through emotion dysregulation.

## Graphical abstract

**Graphical abstract:**
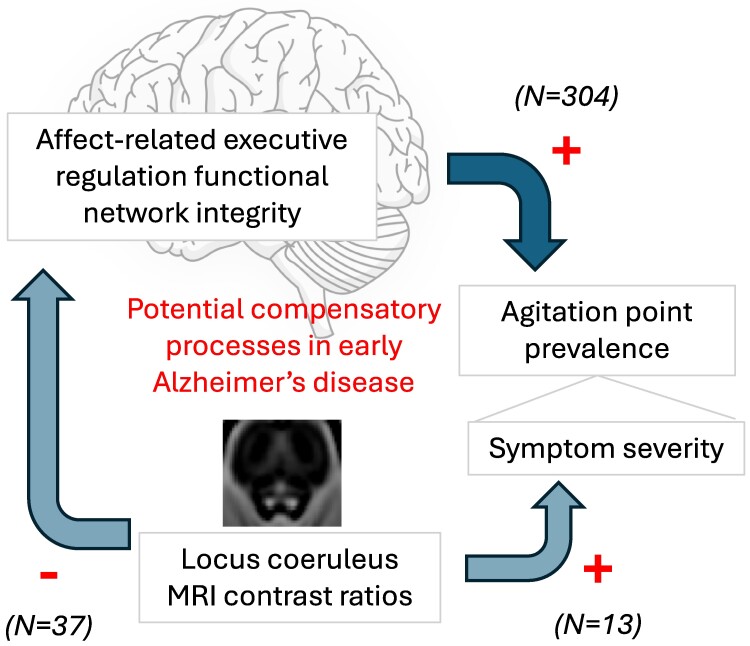

## Introduction

Agitation is a common and difficult-to-treat neuropsychiatric symptom for which there are limited pharmacological treatment options. It is commonly associated with Alzheimer’s disease (AD)-related neuropathology,^1,2^ which underlies the majority of diagnosed dementia cases. Although cross-sectional studies show that agitation point prevalence increases with AD severity,^1,3^ longitudinal studies suggest that agitation severity within an individual increases more over time than does the proportion of patients with agitation.^3,4^ Thus, agitation risk varies among individuals^3,4^ and can be related to factors other than cognitive status or dementia severity. For instance, agitation in AD has been linked to specific structural and functional changes in prefrontal cortex (PFC), anterior cingulate cortex (ACC), insula and amygdala,^2^ as well as higher heart rate variability (HRV),^5^ which raises the possibility of the involvement of aberrant emotion and/or autonomic regulation processes.^6,7^

The locus coeruleus (LC) is a small pontine nucleus that provides widespread noradrenergic innervation to most brain regions. Notably, the LC is also one of the earliest sites affected by AD-related (tau) pathology.^8^ AD-related tau load increases the likelihood of agitation from the earliest stages (odds ratio = 6.1 at Braak stages I–II) when most individuals are still cognitively normal.^9^ Prior to progressive AD-related LC cell death that can be measured from around Braak stages III–IV,^10,11^ early accumulation of hyperphosphorylated tau in the LC can lead to compensatory changes in noradrenergic transmission that aim to maintain normal cognitive and behavioural processes, but can potentially lead to negative consequences in more stressful contexts,^12^ potentially due to an ‘inverted-U-shaped’ relationship between noradrenaline (NA) levels and PFC-dependent cognitive performance.^13^ Functional compensatory overactivity in the LC-NA system could underlie agitation propensity in individuals with symptomatic AD,^14^ and its particular relevance to earlier (preclinical or prodromal) stages of the disease course prior to substantial LC cell loss is supported by recent findings in tau-positive older adults with absent or mild to moderate cognitive impairment. One study reported relatively preserved LC structural integrity (indicated by higher LC MRI contrast ratios, a proxy for LC cell density^15^) in relation to greater impulse dyscontrol domain severity on the Mild Behavioral Impairment Checklist^16,17^; and another study found higher NA metabolism in relation to neuropsychiatric symptoms, including within agitation and disinhibition domains.^18^

The relationship between (compensatory) functional activity, structural brain volume and task performance measures in neurodegenerative conditions may show a non-linear trajectory across the disease course,^19^ and a better understanding of the neural processes underlying agitation could help the development of targeted prevention and treatment strategies, e.g. with noradrenergic medications. There is evidence that enhanced noradrenergic responsiveness in AD is related to agitation and an anti-adrenergic pharmacological approach may be an effective treatment approach^20-22^. To our knowledge, no study has investigated differences in both emotion regulation functional networks and LC structural integrity (MRI contrast ratios) and whether this might be related to agitation in AD. We assessed cross-sectional relationships between these variables in mild cognitive impairment (MCI) and mild AD dementia individuals to test the hypothesis that agitation is driven by impaired emotion regulation, which we predicted would be reflected in dysfunction within an affect-related executive network. In a subsample of individuals with available LC MR imaging data, we also tested whether LC MRI contrast ratios show a positive association with agitation symptom severity, as identified in earlier studies.^16^

## Materials and methods

### Participants

The German Center for Neurodegenerative Diseases (DZNE)-Longitudinal Cognitive Impairment and Dementia (DELCODE) study is a multi-centre, longitudinal, observational study in Germany that enrolled German-speaking healthy controls, MCI and AD dementia patients aged 60 years or older. Participants underwent clinical and neuropsychological testing and imaging procedures including MRI brain scans, and detailed inclusion and exclusion criteria have been described previously.^23^ Patients assessed at baseline as meeting research criteria for amnestic MCI,^24^ or probable AD^25^ were included in the study (*N* = 309). Although the DELCODE study design involved the recruitment of mild AD dementia, defined as Mini Mental State Examination (MMSE) ≥18,^23^ this threshold could be considered to include mild-to-moderate AD dementia,^26^ and three individuals who had recorded baseline MMSE scores of 16 and 17 were included in the analysis. All participants had a study partner available (e.g. spouse, sibling, or child) who could provide third-party medical history.

The DELCODE study was approved by the institutional review boards and ethical committees of each of the participating recruiting sites. All participants provided written informed consent prior to inclusion. DELCODE has been registered with the German Clinical Trials Register (DRKS; https://www.bfarm.de/EN/BfArM/Tasks/German-Clinical-Trials-Register/_node.html; study ID: DRKS00007966).

### Agitation measures

Agitation point prevalence and severity at baseline were obtained from the participants’ study partners, using the Neuropsychiatric Inventory Questionnaire (NPI-Q).^27^ For the Agitation/Aggression NPI-Q item (‘Is the patient resistive to help from others at times, or hard to handle?’), the study partner indicated whether the symptom was present during the past month (‘yes’ or ‘no’) and, if so, rated its severity on a 3-point Likert scale (mild: ‘noticeable, but not a significant change’, moderate: ‘significant, but not a dramatic change’, or severe: ‘very marked or prominent, a dramatic change’).

### Emotion regulation measures

Emotion regulation capacity was indexed by resting-state functional connectivity measures between regions of a proposed affect-related executive regulation network^28^ and a measure of executive performance. Functional connectivity measures were taken between four regions of a distinct fronto-cingular-subcortical circuit proposed to be involved in ‘hot’ executive functions, formed of medial prefrontal cortex (mPFC), ventral ACC, and right/left amygdala, with these specific mPFC and ACC ROIs selected due to their relevance for autonomic function (described further below). As ‘hot’ executive function task measures were not available in the DELCODE dataset, we included the executive function factor score to index overall executive function task performance. This was one of five cognitive domain latent factors generated from confirmatory factor analysis of 27 variables from the DELCODE neuropsychological test battery,^29^ formed of Trail Making Test Parts A and B, Number cancelation test, Symbol Digit Modalities test (oral version) and Flanker task scores, and can be considered an index of ‘cold’ executive function performance.

### MRI acquisition

Structural T1-weighted Magnetization-Prepared Rapid Acquisition Gradient Echo (MPRAGE) and T2*-weighted echo-planar imaging (EPI) resting-state functional MRI (fMRI) scans were obtained using a 3T Siemens scanners and an acquisition protocol that was standardized across all sites^23^ (Table 1). A minority of participants (*N* = 37) underwent an additional scan with a Fast Low Angle Shot (FLASH) sequence optimized to image the LC.

**Table 1:** MRI Scanning parameters

MRI sequence	Matrix size	Slice number; orientation	Voxel size (mm^3^)	Repetition time (TR, ms)	Echo time (TE, ms)	Flip angle (°)	Acquisition time (min:sec)	Additional information
T1/MPRAGE	256 × 256	192; sagittal	1 × 1 × 1	2500	4.33	7	5:08	Inversion time 1100 ms
T2*/EPI	224 × 224 × 165	47; axial	3.5 × 3.5 × 3.5	2580	30	80	7:54	3.5 mm slices, no gap, interweaving
FLASH	320 × 320 × 192		0.75 × 0.75 × 0.75	20	5.56	23	13:50	130 Hz/pixel bandwidth, 7/8 partial Fourier

### Resting-state functional connectivity measures

After excluding two participants’ MPRAGE scans due to poor quality (strong motion or considered unreadable), a total of *N* = 248 had paired EPI resting-state functional and MPRAGE structural data. The images were preprocessed using the CONN toolbox v.20b^30^ default preprocessing pipeline in Statistical Parametric Mapping (SPM12) software (https://www.fil.ion.ucl.ac.uk/spm/). This included realignment, slice-timing correction, and outlier identification using Artifact Detection Tools (https://www.nitrc.org/projects/artifact_detect, which flagged acquisitions with framewise displacement above 0.9 mm or global blood oxygen level–dependent [BOLD] signal changes above 5 SD as potential outliers) of functional data, alongside segmentation of structural data into grey matter, white matter and cerebrospinal fluid (CSF) tissue classes and normalization into standard Montreal Neurologic Institute (MNI) space. Individual structural normalization transformations were applied to co-registered mean functional images, which were smoothed with a Gaussian kernel of 5-mm full-width half maximum. The default sampling resolution of the output was 2 × 2 × 2mm^3^ for the functional and 1 × 1 × 1mm^3^ for the structural images. The default denoising pipeline was then used to remove via linear regression of potential confounding effects, i.e. noise components from WM and CSF areas, estimated rigid-body-motion parameters obtained from realignment, and identified outlier scans or scrubbing, from the BOLD signal. Temporal band-pass filtering was then employed to remove temporal frequencies below 0.008 Hz or above 0.09 Hz from the BOLD signal, to minimize the influence of physiological, head-motion, and other noise sources.

The mPFC mask was a spherical 10-mm region-of-interest (ROI) centred on MNI coordinates (*x* = 2, *y* = 46, *z* = 6), corresponding to a pregenual mPFC region consistently identified from independent literature to be associated with positivity bias and HRV.^6,31,32^ The subgenual ACC mask, defined by the AAL3 atlas,^33^ was used as this region is implicated in autonomic components of emotion.^34^ Right and left amygdala ROI masks were also derived from the Automated Anatomical Labelling 3 (AAL3) atlas.^33^ ROI-to-ROI connectivity *z*-scores were computed for each subject via Fisher’s z-transformation of the pairwise correlations across the entire fMRI time-series (Pearson’s correlation coefficients, *r*), across five pairs of ROIs (mPFC-right amygdala; mPFC-left amygdala, mPFC-ACC, and ACC-right amygdala and ACC-left amygdala).

### Regional grey matter volumes

The MPRAGE structural images were processed using the Computational Anatomy Toolbox (CAT12) (https://neuro-jena.github.io/cat//) in SPM12 software (https://www.fil.ion.ucl.ac.uk/spm/). The CAT12 default voxel-based morphometry (VBM) preprocessing pipeline resulted in normalized and modulated images registered to a template space, segmented into grey matter (GM), white matter (WM) and CSF. Total intracranial volume (TIV) was estimated from the transformation parameters obtained during segmentation. Mean ROI grey matter volumes for each participant were estimated using the Neuromorphometrics atlas (http://Neuromorphometrics.com/). Bilateral regional volume values for amygdala, anterior cingulate cortex, and medial frontal cortex were extracted and divided by TIV for subsequent statistical analyses.

### Locus coeruleus signal intensity measures

We used mean bilateral peak (i.e. maximum) LC MRI contrast ratio values from the FLASH images, given previous findings that differences in peak values were associated with age^35^ and AD-related neuropsychiatric symptoms.^16^ FLASH images were *sinc* interpolated to 0.375 mm^3^ resolution and standardized to a common template using Advanced Normalization Tools (ANTs) v2.1.^36^ The LC and reference masks were delineated in template space as previously described (Betts *et al*., 2017) and warped to each subject in the study-wise template using the ‘WarpImageMultiTransform’ function in ANTs v2.1. Given the use of a group-level LC mask, individual LC masks in each participant’s interpolated FLASH image were visually inspected to ensure no spurious voxels were located in the 4th ventricle or gap in the rostro-caudal axis of the LC. Peak LC MRI contrast ratios were determined relative to reference regions delineated in the rostral pontomesencephalic area using the standard formula as described previously.^35^ The whole LC was divided into thirds along its length to generate the rostral, middle and caudal subregions. Peak LC MRI contrast ratios for the rostral, middle and caudal thirds of the LC were also obtained.

### Statistical analysis

All analyses were performed in R version 4.0.2.^37^ To assess the generalizability of LC analysis outcomes in the LC MRI imaging subgroup (N = 37), we tested for differences in demographic, clinical and neural measures between this subgroup and the whole sample using Welch’s two-sample *t*-test (i.e. assuming unequal variances) for continuous measures and the chi-squared test for independence of proportions.

For the whole sample, we used confirmatory factor analysis (CFA) to test the validity of a latent factor representing affect-related executive regulation network integrity, comprised of the five resting-state functional connectivity measures and the executive function factor score. Variables were all scaled to have mean of 5 and standard deviation (SD) of 2 to optimize model identification, and theoretically plausible modification indices were examined to optimize model fit. As the hypothesized directionality of effects was that impaired emotion regulation drives agitation, we regressed agitation point prevalence or severity onto this latent factor in a structural equation model (SEM).

Since only 37 participants had available LC MRI data, there were likely to be insufficient observations to reliably interpret parameters between the affect-related executive regulation latent factor and LC MRI contrast ratios in an SEM, as at least 5–10 observations per model parameter are recommended.^38,39^ We thus extracted individual latent factor scores and examined their association with LC MRI contrast ratio values in a linear regression model. In an exploratory analysis, we regressed LC MRI contrast ratios on each individual variable that formed the latent factor to examine whether any showed independent associations with LC. Finally, we regressed agitation point prevalence or severity onto LC MRI contrast ratio values.

For all regression models, we controlled for the effects of age and clinical disease severity, represented by higher values on the CDR-SB score, by additionally regressing these covariates onto agitation point prevalence or severity. For regression models that included any resting-state functional connectivity variables, we also controlled for grey matter volume and years of education, as these can be related to functional activity.^40^ In the SEMs, grey matter volume was represented by a latent factor formed of mPFC, amygdala, and ACC TIV-adjusted grey matter volumes, and the individual grey matter volume latent factor scores were extracted for use in simple regression models.

The statistical significance of individual regression paths of interest was formally assessed using the likelihood ratio test (LRT), which compares the fit of a model with the parameter freely estimated to a nested model with the same parameter fixed to zero. Model fit was evaluated using the root-mean-square error of approximation (RMSEA), the comparative fit index (CFI), and the standardized root mean residual (SRMR), with good model fit defined as RMSEA < 0.06 (acceptable: 0.06–0.08), CFI > 0.95 (acceptable: 0.90–0.95) and SRMR < 0.08 (acceptable: 0.08–0.10).^41^ All models were estimated using the lavaan package, version 0.6–12, in R version 3.5.161, using all available data via full information maximum likelihood estimation (FIML) and the robust maximum likelihood estimator with a Yuan–Bentler scaled test statistic (MLR),^42^ apart from models of agitation point prevalence which were estimated using the weighted least squares with mean and variance adjustment (WLSMV) estimator that was more suited to binary variables.

## Results

### Descriptive statistics

Table 2 shows participants’ demographic and clinical characteristics and descriptive statistics for the agitation, emotion regulation, and LC signal intensity variables, and the extent of missing observations. No significant differences in measures or proportions between the whole sample and the LC subsample were observed, apart from lower ACC-right amygdala functional connectivity in the LC subsample. Statistically significant pairwise Pearson’s r correlation coefficients between the analysed variables are shown in Supplementary Table 1.

**Table 2:** Descriptive statistics for the LC, emotion regulation and agitation variables, and demographic characteristics

Variable	Mean (SD), range, or Proportion (*M* = missing)	
	Whole sample (*n* = 309)	LC subsample (*n* = 37)
Age in years	81.1 (6.0), 66.8–96.0	79.8 (6.1), 67.7–92.5
Female (%)	156/309 (51)	18/37 (49)
Education years	13.6 (3.2), 6–20	13.7 (2.7), 8–20
CDR-SB score	2.79 (2.21), 0–12 (M = 5)	2.32 (1.69), 0.5–6.5
Diagnosis	(M = 1)^a^	
MCI	182/308 (40.7)	26/37 (70.3)
Mild AD dementia	126/308 (59.2)	11/37 (29.7)
Agitation present (%)	105/307 (34.2) (M = 2)	14/37 (37.8)
Agitation severity	(M = 1)	(M = 1)
Mild	57/104 (54.8)	7/14 (50)
Moderate	44/104 (42.3)	6/14 (43)
Severe	3/104 (2.9)	0/14
EXEC factor score	−1.03 (0.99), −3.39–1.14 (M = 1)	−0.78 (0.90), −2.35–0.57
Resting-state FC	(M = 61)	(M = 2)
mPFC-L amygdala	0.03 (0.19), −0.40–0.53	0.03 (0.20), −0.25–0.48
mPFC-R amygdala	0.04 (0.18), −0.46–0.50	0.02 (0.16), −0.28–0.40
mPFC-ACC	0.30 (0.20), −0.30–0.96	0.31 (0.13), 0.11–0.59
ACC-L amygdala	0.06 (0.18), −0.45–0.52	0.07 (0.15), −0.22–0.35
ACC-R amygdala^b^	0.05 (0.18), −0.41–0.55	−0.008 (0.14), −0.29–0.30
LC Peak signal intensity ratio	(M = 272)	
Whole	–	0.21 (0.07), 0.10–0.36
Rostral subregion	–	0.16 (0.05), 0.06–0.26
Middle subregion	–	0.18 (0.07), 0.09–0.36
Caudal subregion	–	0.18 (0.07), 0.05–0.33

^a^One participant had missing values for MCI and AD diagnosis data (MMSE = 29, CDR-SB = 0.5) so was treated as missing here but was included in the main analysis.

^b^Only this variable showed a significant difference between the whole sample and LC subsample (*t* = −2.2, df = 51, *P* = 0.03).

### Affect-related executive regulation and agitation

**Figure 1:**
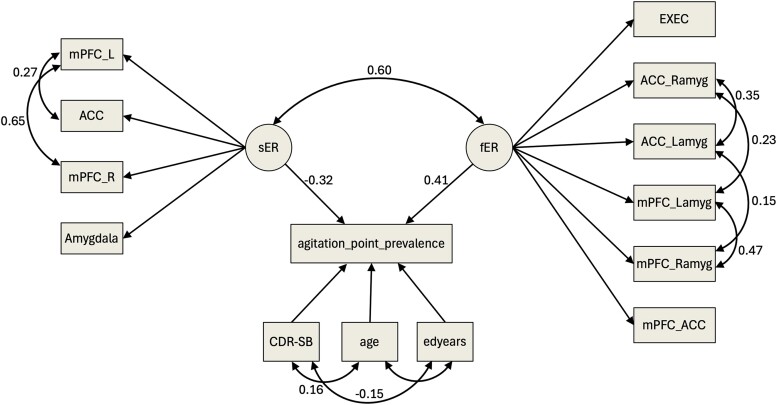
**SEM to assess regression of agitation point prevalence on the affect-related executive regulation latent factor (fER).** The affect-related executive regulation latent factor (fER) was formed of resting-state functional connectivity measures between mPFC-ACC (mPFC_ACC), mPFC-right amygdala (mPFC_Ramyg), mPFC-left amygdala (mPFC_Lamyg), ACC-left amygdala (ACC_Lamyg), ACC-right amygdala (ACC_Ramyg), and executive function factor scores (EXEC). A corresponding structural latent factor (sER) was formed of grey matter volumes of right and left mPFC (mPFC_R, mPFC_L) and averaged bilateral ACC and amygdala values. The fER and sER measurement models showed a good fit after incorporating covariance paths (double-headed arrows) between the observed variables based on modification indices. In a structural equation model, the regression path (single-headed arrow) between agitation point prevalence and the fER latent factor was controlled for grey matter volume (sER), age, education years (edyears), and clinical disease severity (CDR-SB). A separate model for agitation severity is shown in Supplementary Fig. 1. Only statistically significant standardized covariance/regression estimates are displayed. Squares/rectangles represent observed variables and circles represent latent factors. Number of observations used: *N* = 304 for the agitation point prevalence SEM and *N* = 244 for the agitation severity SEM.

The initial fit of the affect-related executive regulation latent factor (CFI 0.880, RMSEA 0.092, SRMR 0.046) was improved after incorporating four covariance paths between the MRI functional connectivity variables based on modification indices [CFI 1, RMSEA 0, SRMR 0.024 (‘fER’ in Fig. 1)]. All factor loadings were >0.5, apart from mPFC-ACC (0.18) and the executive function factor score (0.13), but considering their theoretical relevance in emotion regulation and observing that their removal led to model identification issues, we included these in subsequent regression models.

The latent factor representing grey matter volume comprising mPFC, amygdala and ACC grey matter volumes showed a good fit (CFI 0, RMSEA 1, SRMR 0) with all loadings >0.45 (‘sER’ in Fig. 1). Right/left amygdala and right/left ACC grey matter volume values were averaged to simplify the model and optimize fit, as the correlation between these bilateral regions was >0.8 (Supplementary Table 1).

Regression revealed a significant positive relationship between agitation point prevalence and the affect-related executive regulation latent factor (standardized regression coefficient = 0.18, χ^2^_diff_ = 6.73, df_diff_ =1, *P* = 0.009; CFI = 0.997, RMSEA = 0.015, and SRMR = 0.039). A multiple regression model showed that this positive association survived adjustment for age, grey matter volume, clinical disease severity, and education years (Fig. 1), and there was also a significant negative association between the latent grey matter volume factor and agitation point prevalence (*χ*^2^_diff_ =5.87, *df*_diff_ =1, *P* = 0.015). In contrast, the association between agitation severity and the affect-related executive regulation latent factor was non-significant (standardized regression coefficient = −0.14, *χ*^2^_diff_ = 1.49, *df*_diff_ =1, *P* = 0.22) (see Supplementary Fig. 1).

### Affect-related executive regulation and LC signal intensity

There was a significant negative association between rostral and middle LC MRI contrast ratios and individual affect-related executive regulation latent factor scores in adjusted regression models (Table 3). Of the observed variables forming the affect-related executive factor, only mPFC-left amygdala functional connectivity showed a significant negative association with whole, rostral, and middle LC regions (Supplementary Table 2).

**Table 3:** Regression models assessing relationships between agitation and LC signal intensity

LC	Agitation point prevalence (*N* = 37)				Agitation severity (*N* = 14)				Affect-related executive regulation factor score (*N* = 37)			
	Unadjusted		Adjusted		Unadjusted		Adjusted		Unadjusted		Adjusted^a^	
	Log odds (*P*-value)	95% CI	Log odds	95% CI	β (*P*-value)	95% CI	β (*P*-value)	95% CI	β (*P*-value)	95% CI	β (*P*-value)	95% CI
Whole	−4.05 (0.40)	−14.00∼5.13	−3.01 (0.57)	−13.9∼7.19	2.45 (0.45)	−2.00∼6.91	3.15 (0.14)	−1.27∼7.57	**−1.66** (0.026)	−3.11∼−0.20	−1.14 (0.09)	−3.11∼0.25
Rostral	−11.05 (0.11)	−27.00∼−1.73	−10.98 (0.15)	−0.46∼0.45	**8.58** (0.047)	0.13∼17.03	**8.87** (0.045)	0.23∼17.52	**−2.94** (0.003)	−4.84∼−1.04	**−2.66** (0.016)	−4.78∼−0.53
Middle	−3.13 (0.52)	−13.32∼6.04	−2.38 (0.66)	−13.6∼7.88	1.17 (0.60)	−3.65∼6.00	2.47 (0.30)	−2.64∼7.58	**−1.95** (0.008)	−3.36∼−0.54	**−1.80** (0.030)	−3.41∼−0.19
Caudal	−5.27 (0.29)	−15.81∼4.26	−4.30 (0.41)	−0.50∼0.36	4.11 (0.11)	−1.15∼9.37	3.94 (0.12)	−1.28∼9.16	−1.26 (0.12)	−2.81∼0.29	−0.95 (0.26)	−2.64∼0.75

Logistic regression models were used for agitation point prevalence and linear regression models for agitation severity. Adjusted models included age and CDR-SB scores as covariates. Abbreviations: CI = confidence interval, FC = functional connectivity, LC = locus coeruleus (peak signal intensity). Statistically significant regressions shown in bold.

^a^In addition to age and clinical disease severity, this model was also adjusted for structural volume (via the grey matter latent factor scores) and education years.

### LC signal intensity and agitation

There was no significant association between LC MRI contrast ratios and agitation point prevalence at study baseline, but in individuals who had agitation (*N* = 14 of 37), higher rostral LC MRI contrast ratio values were related to greater agitation severity, over and above the effects of age and clinical disease severity (Table 3).

As there were significant correlations between age, CDR-SB and grey matter volume, and between CDR-SB and years of education (Supplementary Table 1), we calculated the variance inflation factor (VIF) values for covariates in simple adjusted regression models, which showed no or low multicollinearity (Supplementary Table 3).

## Discussion

In this study of individuals with amnestic MCI and mild AD dementia, agitation point prevalence showed a positive relationship with a latent factor representing the functional integrity (and a negative relationship with a corresponding structural measure) of a proposed affect-related executive regulation network. It is possible that compensatory neuronal processes in the presence of structural loss may lead to varying degrees of adaptive capacity depending on the level of contextual demand. In more stressful contexts, higher compensatory functional activity might impair self-regulatory capacity and increase agitation propensity.^12,13^ Earlier studies support the concept that baseline LC-NA system responsiveness/capacity may correspond to symptom profile and predict treatment response in AD; i.e. reducing overactivity/responsiveness might improve agitation.^20-22^ In a subsample with LC MRI data, rostral LC MRI contrast ratios were positively associated with agitation severity (*N* = 13), and negatively associated with the latent (functional) affect-related regulation factor scores (*N* = 37). The preliminary finding is consistent with an earlier study^16^ that found higher LC MRI contrast ratios in relation to greater impulse dyscontrol domain severity in an early AD cohort.^16^ Resting-state functional connectivity between the mPFC ROI and left amygdala, which was related to LC MRI contrast ratios, has been reported to reflect implicit emotion regulation processes^32^ and responds to HRV biofeedback.^43^ These findings were not explained by differences in age and clinical disease severity nor in education years or grey matter volumes and are consistent with the hypothesized involvement of the LC-NA system and emotion (and autonomic) dysregulation in agitation in AD, alongside the effects of compensatory processes.

In the context of AD-related LC neurodegeneration, higher resting-state functional connectivity within an affect-related executive regulation network might enhance certain cognitive or emotional processes,^44^ but may impair others,^13^ e.g. reduced adaptive capacity in more demanding contexts.^45^ Compensatory functional connectivity in emotion regulation regions in AD is supported by earlier findings of higher resting HRV (a putative index of the functional capacity of PFC regions supporting self-regulatory processes^7^) in association with greater AD severity in older cognitively normal and MCI individuals, which was mediated by increased (compensatory) fMRI activation in ACC.^46^ Higher HRV has also been associated with greater agitation risk in AD dementia.^5^

Although not all model parameters were statistically significant, the regressions of LC MRI contrast ratios and the latent factor on agitation severity versus point prevalence were of opposing signs, which points to a possible dissociation between the mechanisms underlying agitation *risk* and *severity*. One possible explanation is that this pattern might be related to dysfunction at different levels of an emotion regulation hierarchy,^47^ where impaired early and fast (amygdala) appraisal mechanisms predispose certain AD individuals to develop agitation, whilst impaired late and slow (PFC) appraisal mechanisms in these individuals contribute to greater symptom severity. Alternatively, it could relate to the two major apparent influences on individual differences in LC MRI contrast ratios: neurodegeneration in aging and disease that reduces LC MRI contrast and leads to positive correlations between LC MRI contrast and cognition in older adults,^48,49^ versus high tonic noradrenergic activity that underlies negative relationships between LC MRI contrast and HRV^50,51^ and positive correlations between LC functional activity measures and anxiety/stress disorders.^52-54^ The current findings would be consistent with LC neurodegeneration levels predicting point prevalence of agitation and tonic noradrenergic hyperactivity predicting agitation severity.

### Limitations

The LC analyses were limited by the small sample size of the group who had LC MRI data (*N* = 37, and of these, *N* = 14 had agitation point prevalence and *N* = 13 had agitation severity data). Future studies with larger sample sizes could employ other approaches to investigate whether the relationship between emotion regulation and LC MRI contrast ratios differs by agitation status e.g. in a multigroup SEM. The association between rostral LC MRI contrast ratios and agitation severity did not persist after including middle and caudal LC MRI contrast ratios as covariates, as the analysis was likely underpowered to show a regionally specific effect. Although LC MRI signal contrast in older adults reflects the density of neuromelanin-containing LC neurons,^15^ it does not directly reflect LC activity and questions remain about the precise mechanism of the LC MRI contrast^55^ and its temporal relationship to LC neurodegeneration.^56^ The relatively novel T1-weighted FLASH sequence approach used to obtain LC MRI contrast ratios has been less frequently employed and may yield lower LC contrast values compared to other sequences, which may have influenced the findings. We did not perform unwarping of functional MRI data using fieldmaps, which may have led to greater susceptibility-induced distortions, although the relative impact on larger (i.e. mPFC, amygdala and ACC) ROIs may be smaller. The 8-min scan duration may have limited the reliability of fMRI data compared to longer scan durations.^57^

The hypothesized directionality of effects, i.e. impaired LC function predicting emotion regulation, and impaired emotion regulation or LC function predicting agitation, cannot be tested using cross-sectional data and there may be bidirectional relationships or alternative mechanisms that were not accounted for in our analyses. For example, older adults can show higher-resting-state connectivity in corticolimbic networks following negative emotional episodes, which may represent a poor recovery mechanism.^58^ Resting-state connectivity measures can also be context dependent^59^ and it would be informative to compare resting versus task-based functional connectivity analyses of emotion regulation networks. The models employed, including simple regression and SEMs, assumed linear relationships between variables, which may not have fully captured potential non-linear associations between measures of LC-NA integrity, emotion regulation and agitation in AD.

Agitation measured using the NPI-Q Agitation/Aggression subscale relies on a binary answer (Yes/No) to a single question, which may have limited the detection of the clinical syndrome as, particularly in less severe clinical AD stages, agitation may present more subtly, e.g. as irritability. Also, the NPI-Q does not measure symptom frequency or associated distress, which can be considered to contribute to clinical severity, and other approaches to measuring agitation should be explored in future studies. The MCI and mild AD dementia participants were clinically diagnosed without available AD biomarker confirmation, so it is possible that this led to a degree of mismatch between AD neuropathology levels and clinical status. The extent to which other non-AD neuropathologies potentially contributed to any cognitive impairment is also unclear.

We included both neural and executive function measures to represent the coordinated activity of the proposed affect-related executive regulation network via a latent factor, which showed statistical validity in terms of model fit, but it would be important to assess its generalizability by replicating the analyses in other datasets. The executive function factor scores could be considered an index of ‘cold’ executive function, and other ‘hot’ executive function task performance measures, if they had been included in the DELCODE dataset, may have more optimally represented cognitive processes underlying agitation propensity. The executive function factor scores showed a low loading (0.13) onto the affect-related executive regulation latent factor, and its inclusion contributed only modestly to increasing the variance explained in the latent factor (*R*^2^ from 0.13 to 0.137). On the other hand, this suggests it still provides some unique information and may capture aspects of affect-related executive regulation that are not adequately explained by neural connectivity measures alone.

In summary, our findings implicate the involvement of LC and emotion dysregulation in agitation in AD and support the presence of compensatory processes. Further studies are needed to replicate and build on these findings, incorporating longitudinal designs, measures of autonomic function, non-linear and mediation modelling approaches to explore potential causal and context-dependent relationships across AD severity stages.

## Supplementary Material

fcae457_Supplementary_Data

## Data Availability

The DELCODE study data, which support this study, are not publicly available, but may be provided by the DELCODE study committee based on individual data-sharing agreements (see https://www.dzne.de/en/research/studies/clinical-studies/delcode/). The R code that supports the SEM analyses within this paper are available at https://github.com/k-y-liu/DELCODE_agitationLC.
