# Supplementary material for: Locus coeruleus signal intensity and emotion regulation in agitation in Alzheimer’s disease
Source: Brain Commun. 2024 Dec 17;7(1):fcae457. doi: 10.1093/braincomms/fcae457 (PMC11724426; doi:10.1093/braincomms/fcae457)
Supplement: fcae457_Supplementary_Data [file fcae457_supplementary_data.docx]

SUPPLEMENTARY MATERIAL

**Locus coeruleus signal intensity and emotion regulation in agitation in Alzheimer’s disease**

[Supplementary Figure 1: SEM to assess regression of agitation severity on the affect-related executive regulation latent factor (fER). 2](#_Toc168931643)

[Supplementary Table 1: Correlation matrix showing pairwise (Pearson’s r) correlations between analysed variables. 3](#_Toc168931644)

[Supplementary Table 2: Associations between LC MRI contrast ratios and mPFC-L amygdala functional connectivity. 5](#_Toc168931645)

[Supplementary Table 3: Regression coefficients for covariates and parameters of multicollinearity in adjusted models in Table 3 6](#_Toc168931646)

### **Supplementary Figure 1: SEM to assess regression of agitation severity on the affect-related executive regulation latent factor (fER).**


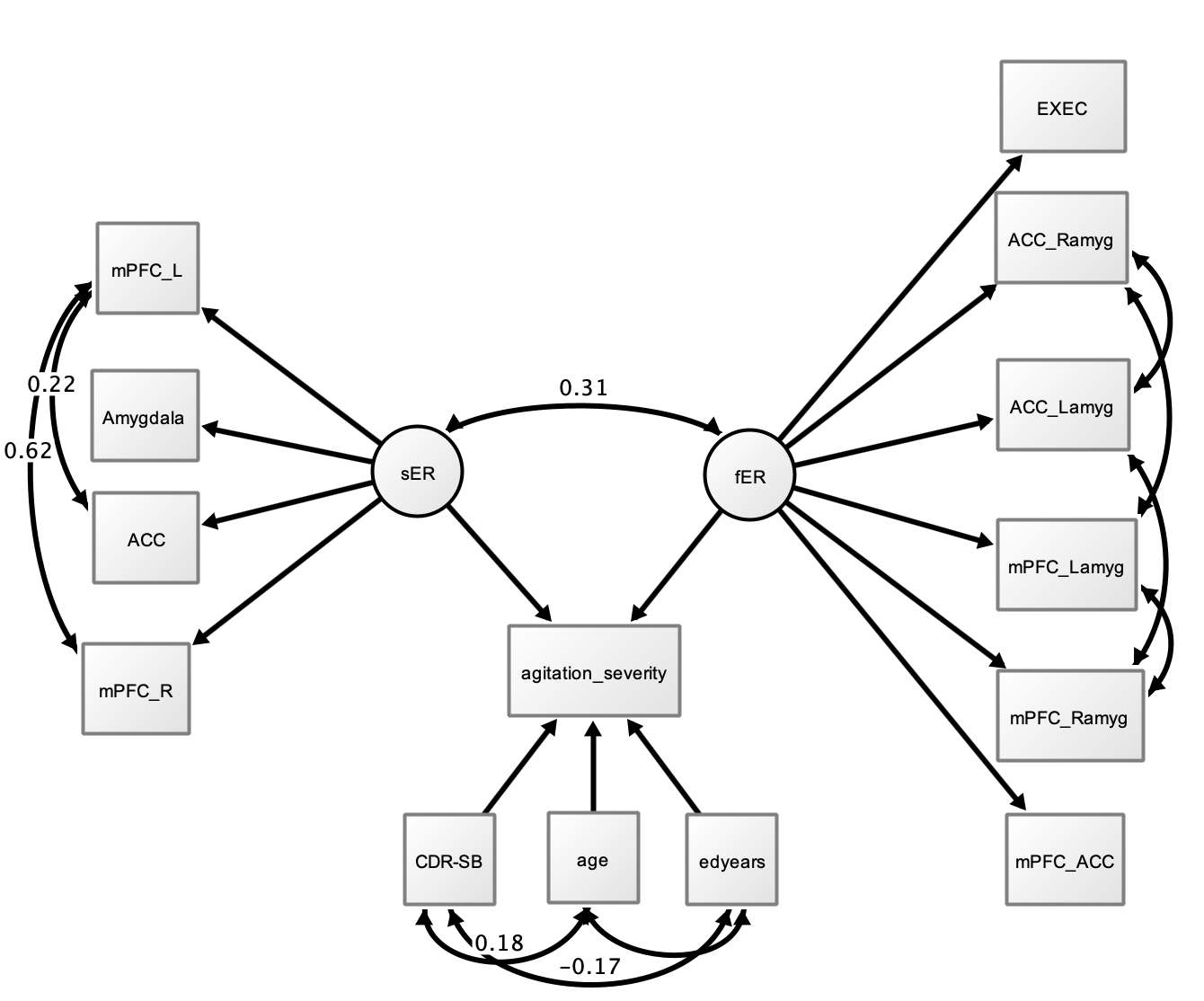


The affect-related executive regulation latent factor (fER) was formed of resting state functional connectivity measures between mPFC-ACC (mPFC_ACC), mPFC-right amygdala (mPFC_Ramyg), mPFC-left amygdala (mPFC_Lamyg), ACC-left amygdala (ACC_Lamyg), ACC-right amygdala (ACC_Ramyg), and executive function factor scores (EXEC). A corresponding structural latent factor (sER) was formed of grey matter volumes of right and left mPFC (mPFC_R, mPFC_L) and averaged bilateral ACC and amygdala values. The fER and sER measurement models showed good fit after incorporating covariance paths (double-headed arrows) between the observed variables based on modification indices. In a structural equation model, the regression path (single-headed arrow) between agitation point prevalence and the fER latent factor was controlled for grey matter volume (sER), age, education years (edyears), clinical disease severity (CDR-SB). Only statistically significant standardized covariance/regression estimates are displayed. Squares/rectangles represent observed variables and circles represent latent factors. Number of observations used: N=244.

### **Supplementary Table 1: Correlation matrix showing pairwise (Pearson’s r) correlations between analysed variables.**

|  | 1 | 2 | 3 | 4 | 5 | 6 | 7 | 8 | 9 | 10 | 11 | 12 | 13 | 14 | 15 | 16 | 17 | 18 | 19 | 20 | 21 |
| --- | --- | --- | --- | --- | --- | --- | --- | --- | --- | --- | --- | --- | --- | --- | --- | --- | --- | --- | --- | --- | --- |
| 1 | 1 |  |  |  |  |  |  |  |  |  |  |  |  |  |  |  |  |  |  |  |  |
| 2 | .52 | 1 |  |  |  |  |  |  |  |  |  |  |  |  |  |  |  |  |  |  |  |
| 3 | .14 |  | 1 |  |  |  |  |  |  |  |  |  |  |  |  |  |  |  |  |  |  |
| 4 | .43 | .24 |  | 1 |  |  |  |  |  |  |  |  |  |  |  |  |  |  |  |  |  |
| 5 | .29 | .37 |  | .40 | 1 |  |  |  |  |  |  |  |  |  |  |  |  |  |  |  |  |
| 6 | .13 |  | .13 |  |  | 1 |  |  |  |  |  |  |  |  |  |  |  |  |  |  |  |
| 7 | .16 |  | .15 | .14 |  | .28 | 1 |  |  |  |  |  |  |  |  |  |  |  |  |  |  |
| 8 |  |  |  |  |  | .18 | .71 | 1 |  |  |  |  |  |  |  |  |  |  |  |  |  |
| 9 |  |  |  | .18 |  | .28 | .37 | .29 | 1 |  |  |  |  |  |  |  |  |  |  |  |  |
| 10 | .16 |  |  | .18 |  | .30 | .42 | .30 | .81 | 1 |  |  |  |  |  |  |  |  |  |  |  |
| 11 | .18 |  | .18 | .16 |  | .22 | .40 | .37 | .36 | .46 | 1 |  |  |  |  |  |  |  |  |  |  |
| 12 |  |  | .19 | .15 |  | .20 | .34 | .43 | .42 | .37 | .80 | 1 |  |  |  |  |  |  |  |  |  |
| 13 | -.46 |  |  |  |  |  |  |  |  |  |  |  | 1 |  |  |  |  |  |  |  |  |
| 14 | -.49 |  |  |  | -.34 |  |  |  |  |  |  |  | .76 | 1 |  |  |  |  |  |  |  |
| 15 | -.49 |  |  |  |  |  |  |  |  |  |  |  | .91 | .66 | 1 |  |  |  |  |  |  |
| 16 | -.37 |  |  |  |  |  |  |  |  |  |  |  | .88 | .76 | .72 | 1 |  |  |  |  |  |
| 17 |  |  |  |  |  | .29 |  |  |  |  |  |  |  |  |  |  | 1 |  |  |  |  |
| 18 | -.13 |  | -.21 | -.14 |  | -.25 | -.19 | -.20 | -.33 | -.28 | -.24 | -.32 |  |  |  |  |  | 1 |  |  |  |
| 19 | -.15 | -.15 | -.14 |  |  | -.50 | -.34 | -.19 | -.37 | -.36 | -.27 | -.25 |  |  | .37 |  | -.17 | .18 | 1 |  |  |
| 20 | .90 | .65 | .19 | .55 | .65 | .15 | .15 |  |  | .17 | .18 |  | -.36 | -.47 | -.43 |  |  | -.11 | -.17 | 1 |  |
| 21 | .20 |  | .20 | .22 |  | .34 | .72 | .54 | .78 | .81 | .75 | .73 |  |  |  |  |  | -.35 | -.43 | .19 | 1 |
| 22 |  |  |  |  |  |  |  |  |  |  | -.24 | -.27 |  | .56 |  |  |  |  |  |  | -.23 |

**1.**mPFC-left amygdala rsFC; **2.** mPFC-right amygdala rsFC; **3.** mPFC-ACC rsFC; **4.** ACC-left amygdala rsFC; **5.** ACC-right amygdala rsFC, **6.** Executive function factor score (EXEC); **7.** Right mPFC grey matter volume; **8.** Left mPFC grey matter volume; **9.** Left amygdala grey matter volume; **10.** Right amygdala grey matter volume; **11.** Right ACC grey matter volume; **12.** Left ACC grey. Matter volume; **13.** Whole LC peak MRI contrast ratio; **14.** Rostral LC peak MRI contrast ratio; **15.** Middle LC peak MRI contrast ratio; **16.** Caudal LC peak MRI contrast ratio; **17.** Education years; **18.** Age; **19.** CDR-SB score; **20.** Latent affect-related executive regulation network factor score; **21.** Latent grey matter volume factor score; **22.** Agitation severity.

Abbreviations: ACC, anterior cingulate cortex; CDR-SB, Clinical Dementia Rating-Sum of Boxes; LC, locus coeruleus; mPFC, medial prefrontal cortex; rsFC, resting state functional connectivity.

Only significant (p<0.05) correlations are shown.

### **Supplementary Table 2: Associations between LC MRI contrast ratios and mPFC-L amygdala functional connectivity.**

| LC | mPFC-L amygdala resting state functional connectivity | | | |
| --- | --- | --- | --- | --- |
|  | Unadjusted | | Adjusted^a^ | |
|  | 𝛽 | 95% CI | 𝛽 | 95% CI |
| Whole | **-1.25**** | -2.10~-0.39 | **-1.02*** | -1.93~-0.10 |
| Rostral | **-1.83**** | -2.98~-0.68 | **-1.53*** | -2.71~-0.36 |
| Middle | **-1.33**** | -2.16~-0.50 | **-1.19*** | -2.07~-0.31 |
| Caudal | **-1.06*** | -2.00~-0.12 | -0.84 | -1.78~-0.09 |

Of the observed variables forming the affect-related executive regulation latent factor, only mPFC-L amygdala functional connectivity showed statistically significant associations with LC MRI contrast ratios. Statistically significant regressions shown in bold, * *p* < 0.05, ** p<0.01.

^a^ In addition to age and clinical disease severity, this model was also adjusted for structural volume (via individual grey matter latent factor scores) and education years.

### **Supplementary Table 3: Regression coefficients for covariates and parameters of multicollinearity in adjusted models in Table 3**

| Covariate | Agitation point prevalence (N=37) | | | | Agitation severity (N=14) | | | | Affect-related executive regulation factor score (N=37) | | | |
| --- | --- | --- | --- | --- | --- | --- | --- | --- | --- | --- | --- | --- |
|  | Whole LC | Rostral LC | Middle LC | Caudal LC | Whole LC | Rostral LC | Middle LC | Caudal LC | Whole LC | Rostral LC | Middle LC | Caudal LC |
| Age | -0.04 (0.48), 1.13 | -0.03 (0.15), 1.05 | -0.05 (0.42), 1.10 | -0.05 (0.42), 1.10 | 0.05 (0.10), 1.00 | 0.04 (0.17), 1.06 | 0.05 (0.10), 1.02 | 0.05 (0.10), 1.01 | -0.02 (0.05), 1.58 | -0.02 (0.09), 1.63 | **-0.02** (0.04), 1.56 | -0.02 (0.06), 1.60 |
| CDR-SB | -0.04 (0.87), 1.21 | -5.7e-05 (1.0), 1.10 | -0.04 (0.86), 1.26 | -0.04 (0.86), 1.26 | -0.07 (0.41), 1.15 | -0.08 (0.29), 1.14 | -0.07 (0.47), 1.24 | 0.002 (0.98), 1.05 | -0.04 (0.25), 1.41 | -0.04 (0.29), 1.39 | -0.03 (0.38), 1.47 | -0.06 (0.13), 1.31 |
| Grey matter volume factor score |  |  |  |  |  |  |  |  | -0.10 (0.21), 1.60 | -0.07 (0.33), 1.56 | -0.09 (0.21), 1.58 | -0.07 (0.33), 1.56 |
| Education years |  |  |  |  |  |  |  |  | -0.003 (0.88), 1.19 | 0.008 (0.72), 1.25 | -0.001 (0.96), 1.19 | -0.004 (0.87), 1.19 |

Values displayed are Log odds or 𝛽 (p-value), variance inflation factor (VIF). Statistically significant (p<0.05) values are shown in bold. VIF values ranged between 1.00 to 1.63), indicating no or low multicollinearity.
